# Intramedullary spinal cord cavernous malformations—association between intraoperative neurophysiological monitoring changes and neurological outcome

**DOI:** 10.1007/s00701-022-05354-z

**Published:** 2022-09-06

**Authors:** Sebastian Niedermeyer, Andrea Szelenyi, Christian Schichor, Joerg-Christian Tonn, Sebastian Siller

**Affiliations:** Department of Neurosurgery, LMU Hospital, Ludwig-Maximilian-University Munich, Marchioninistrasse 15, 81377 Munich, Germany

**Keywords:** Spinal cavernoma, Intraoperative neurophysiological monitoring, McCormick score, Microsurgical resection

## Abstract

**Background:**

Microsurgical resection of spinal cord cavernous malformations can be assisted by intraoperative neurophysiological monitoring (IONM). While the clinical outcome after surgical resection has been discussed in several case series, the association of intraoperative IONM changes and detailed neurological outcome, however, has not been analyzed so far.

**Methods:**

Seventeen patients with spinal cavernomas underwent surgery between 02/2004 and 06/2020. Detailed neurological and clinical outcome as well as IONM data including motor-evoked potential (MEP) and somatosensory-evoked potential (SSEP) monitoring were retrospectively analyzed. Intraoperative IONM changes were compared to outcome at 3-month and 1-year follow-up in order to identify surrogate parameters for an impending neurological deficit.

**Results:**

Compared to the preoperative state, McCormick score at 1-year follow-up remained unchanged in 12 and improved in five patients, none worsened, while detailed neurological examination revealed a new or worsened sensorimotor deficit in 4 patients. The permanent 80% amplitude reduction of MEP and 50% amplitude reduction of SSEP showed the best diagnostic accuracy with a sensitivity of 100% and 67% respectively and a specificity of 73% and 93% respectively. The relative risk for a new neurological deficit at 1-year follow-up, when reversible IONM-deterioration was registered compared to irreversible IONM deterioration, was 0.56 (0.23–1.37) for MEP deterioration and 0.4 (0.18–0.89) for SSEP deterioration.

**Conclusions:**

Reversible IONM changes were associated with a better neurological outcome at follow-up compared to irreversible IONM deterioration during SCCM surgery. Our study favors the permanent 80% amplitude reduction criterion for MEP and 50% amplitude reduction criterion for SSEP for further prospective evaluation of IONM significance and the effectiveness of corrective maneuvers during SCCM surgeries.

**Supplementary Information:**

The online version contains supplementary material available at 10.1007/s00701-022-05354-z.

## Introduction

Vascular lesions of the spinal cord account for about 6–7% of all spinal tumors [30], while spinal cord cavernous malformations (SCCM) comprise only 5–12% of these spinal vascular lesions [3, 4, 8]. SCCM occur in a sporadic as well as in a familial/syndromal form and can remain clinically asymptomatic. If they become symptomatic, acute onset with recurrent and/or progressive neurological deficits may occur due to bleeding. Sensory or motor disturbances are most frequent (about 60% respectively) followed by pain (30%) and bladder/bowl dysfunction (20%) [3]. The annual risk of the first hemorrhage is approximately 2.1% per year [3], but reaches 6.3% (3.0–13.2%) risk of symptomatic recurrent hemorrhage per person year [40].

While therapeutic strategy still depends on neurosurgeons’ preference and conservative treatment concepts have been described for asymptomatic lesions [12, 14], surgical (gross-total) resection is still the most definitive therapy of symptomatic SCCM and is able to eliminate the associated subsequent lifelong hemorrhagic risk [3, 22, 31, 32, 42]. The clinical presentation, surgical management, and selection of surgical approaches as well as long-term outcome after surgical resection of SCCM have been discussed in various studies [3, 20, 22, 31, 32].

Only few case series have additionally suggested intraoperative neurophysiological monitoring (IONM) to be a reasonable surgical adjunct for SCCM resection for prevention of iatrogenic injury to spinal cord fiber tracts or nerve roots with the consequence of postoperative neurological deterioration [1, 3, 20, 22, 26, 31, 35]. However, a corresponding case series describing microsurgical SCCM resection with stringent utilization of multimodal IONM including both somatosensory-evoked potential (SSEP) and motor-evoked potential (MEP)—as generally recommended in IMSCT surgeries [35, 39, 41]—is still lacking. Moreover, due to their biology, resection of vascular lesions like SCCM have to be considered separately from resection of infiltrating intramedullary tumor lesions like astrocytoma or ependymomas and, therefore, drawing a simple analogy for the impact of IONM in spinal cord astrocytoma/ependymoma resection to SCCM resection is not accurate.

The aim of our study was therefore to closely describing the association of IONM changes and detailed postoperative neurological status at short- and long-term follow-up in intramedullary SCCM surgery with state-of-the-art IONM.

## Methods

This retrospective study is a case collection of seventeen patients that underwent surgery for SCCM at the Neurosurgical Department, University of Munich (LMU, Germany), between February 2004 and June 2020. After study approval by the local Institutional Review Board (AZ19-569), the patients’ medical records and radiological studies were retrospectively reviewed. Despite the long inclusion period of our study, the treatment regime and surgical management incl. IONM, except for D-wave monitoring, as well as imaging/clinical evaluation were standardized and homogenous for all cases. Inclusion criteria were histological diagnosis of cavernous malformation and stringent use of intraoperative neurophysiological monitoring.

## Imaging evaluation

The size of SCCM was defined as the largest measurement in either dimension (axial, sagittal, coronary). Evaluation of tumor location and distribution were based on preoperative contrast-enhanced magnetic-resonance imaging (MRI) as well as on intraoperative findings: On the basis of radiographical and intraoperative observations, lesions were characterized as either dorsal or ventral of the coronary spinal cord midline or as central.

## Clinical evaluation

Detailed neurological assessment and clinical evaluation according to the classification of McCormick (MCS) [25] was performed for all patients at admission to the hospital, at discharge, at 3-month and at 1-year follow-up examination. Detailed neurological assessment included cranial nerve examination, examination of motor system according to the MRC (Medical Research Council) [27], and sensory function as well as assessment of gait.

## Surgical treatment regimen and intraoperative neurophysiological monitoring

All the surgically treated patients were operated on at least 2 weeks after onset of symptoms after neurological stabilization and follow-up MRI.

Microsurgical tumor resection was performed under ultrasound-guidance and continuous multimodal IONM of both somatosensory-evoked potentials (SSEP) and motor-evoked potentials (MEP). Recording from segmental target muscles for all relevant myotomes simultaneously insured that all pertinent motor tracts/fibers were monitored. For stimulating and recording, an integrated IONM system is used (EWACS-System until 2012, since then ISIS, both Inomed Co., Emmendingen/Germany). To elicit MEP recording, stimulating electrodes are positioned over C1, C2, C3, and C4 (according to the International 10–20-EEG-system) to achieve far lateral or midline interhemispheric stimulation. For transcranial electric stimulation (TES), constant current anodal stimulation consisting of a train of consecutive five pulses (3–7) with an interstimulus interval of 4 ms was applied. Before 2016, a biphasic fashion with a total duration of the individual pulse width of 1.2–1.6 ms, and a maximum intensity of 150 mA at 300 V; since 2016, monophasic stimulation with an individual pulse width of 0.5 ms and a maximum intensity of 250 mA at 400 V was used. TES was set recording the maximum of MEP of those muscles caudally to the caudal tumor level without inducing disturbing body movements. In case of MEP deterioration, warnings were issued and—if possible—stimulation intensity increased. For recording, stainless steel subdermal electrodes (Inomed Co., Emmendingen, Germany; Spes Medical, Genoa, Italy) are placed bilaterally in thenar, hypothenar, and tibialis anterior muscles, as well as additionally in the biceps, brachioradialis, triceps, iliopsoas, adductor, quadriceps femoris, foot flexor, extensor hallucis longus, and/or sphincter muscle groups according to the spinal level of interest. Since 2016, D-wave recording was performed with an epidural catheter (FSR-3, Inomed Co., Emmendingen, Germany) placed caudal to the lesion site. The same stimulation intensity was used as eliciting leg muscle MEP. SSEP with an individual pulse width of 400 ms, 2.3–5.1 Hz repetition rate, and maximum 40 mA were performed in median and posterior tibial nerve, and average according to the signal–noise-level. After patient positioning, baseline recordings of SSEP and MEP are obtained. SSEP and MEP were continuously recorded in an alternating fashion throughout surgery. If TES-related patient movement was considered disturbing with microdissection, those were performed on surgeons’ request and while pausing dissection. Electrophysiological data were continuously analyzed by a technician trained in IONM and supervised by a senior physician. All procedures were performed with total intravenous anesthesia, avoiding the application of muscle relaxants despite for intubation purposes.

Any reduction of SSEP amplitude and/or an increase of SSEP latency as well as decrement in MEP amplitude, especially during or immediately after surgical manipulation, was immediately issued to the surgical team and checked for relevance excluding technical (e.g., dislocation of electrodes, technical artefact), anesthesiologic (e.g., lowering of blood pressure or body temperature, change of intravenous anesthesia management, or addition of volatile anesthetics) causes, and temporary surgical reasons (e.g., irrigation with cold saline solution). In circumstances of IONM changes, the most recent surgical steps were reconsidered, and immediate corrective actions were stepwise initiated, i.e., modification of the surgical technique with temporary halted resection, reduction of traction on the tumor or surrounding tissue, irrigation with warm saline solution, application of high-dose prednisolone, and/or continuation of resection at distant sites. Every patient received 1 g of methylprednisolone before skin incision.

## Data interpretation

For analysis of association between IONM changes and detailed neurological outcome, the complete IONM dataset was retrospectively reviewed by an expert senior neurophysiologist who was blinded for the neurological/clinical outcome. To determine which criterion was most efficient in detecting a new postoperative deficit and therefore could be called a “warning criterion,” we retrospectively tested different permanent IONM events as warning criteria (Tables 6 and 2) for an impending long-term neurological deficit. IONM events were additionally classified as either “transient deteriorated” (complete resolution within surgery till dura closure) or “permanent deteriorated” (persistence after dura closure). If IONM changes were “permanent deteriorated” and the patient exhibited a new or worsened neurological deficit matching the modality of IONM (i.e., sensory changes, ataxia, and/or pain with regard to SSEP; or motor deficits and/or spasticity with regard to MEP), these changes were called “true positive” [31], respectively, and “false positive” (FP) if the patient did not exhibit new or worsened neurological deficits. If MEP and/or SSEP remained stable or changed only transiently, but the patient showed neurological deterioration, the result was “false negative” [19]. If MEP or SSEP remained stable or changed transiently and no new or no aggravated symptoms occurred postoperatively, the result was “true negative” (TN) (Table 1).

**Table 1:** Calculation of diagnostic and surrogate test (based on Holdefer et al. [9])

IONM deterioration	No new deficit	New deficit	Diagnostic test	Surrogate test
None	a (TN)	b (FN)	$\mathrm{Sensitivity}=\frac{\mathrm{f}}{\mathrm{b}+\mathrm{d}+\mathrm{f}}$	$\mathrm{Reversible risk}=\frac{\mathrm{d}}{\mathrm{c}+\mathrm{d}}$
Transient	c (TN)	d (FN)	$\mathrm{Specificity}=\frac{a+c}{\mathrm{a}+\mathrm{c}+\mathrm{e}}$	$\mathrm{Irreversible risk}=\frac{\mathrm{f}}{\mathrm{e}+\mathrm{f}}$
Permanent significant	e (FP)	f (TP)	$\mathrm{PPV}=\frac{\mathrm{f}}{\mathrm{e}+\mathrm{f}}$	$\mathrm{Relative risk}=\frac{\mathrm{reversible risk}}{\mathrm{irreversible risk}}$
			$$\mathrm{NPV}=\frac{\mathrm{a}+\mathrm{c}}{\mathrm{a}+\mathrm{c}+\mathrm{b}+\mathrm{d}}$$	
			$$\mathrm{Likelihood ratio}=\frac{\mathrm{sensitivity}}{1-\mathrm{specificity}}$$	

*IONM* intraoperative neurophysiological monitoring, *TN* true negative, *TP* true positive, *FN* false negative, *FP* false positive, *PPV* positive predictive value, *NPV* negative predictive value

Assessment of diagnostic test performance of IONM including sensitivity, specificity, positive predictive value (PPV), and negative predictive value (NPV) and surrogacy analysis including reversible risk, irreversible risk, and relative risk (RR) were calculated as described in Table 1. For calculation of likelihood ratio and surrogate parameters, a value of 0.5 was added to all cells if one of the cells was zero [9].

## Statistical analysis

Statistical analysis was performed using GraphPad Prism 8 (GraphPad Software, 2365 Northside Dr., Suite 560, San Diego, CA 92,108). Differences were statistically significant if the *p* value was < 0.05. The patient population was described with summary statistics; mean values are reported as the mean ± standard deviation; median values are reported as median (range). For comparison of groups for differences, the Student’s *t*-test was used for numeric values, Mann–Whitney Rank Sum test for ordinal variables, and *χ*^2^-test resp. Fisher’s exact test (in case of 2 × 2-contingency tables) for nominal variables.

## Results

### Patients’ characteristics and clinical presentation

Seventeen patients underwent IONM-assisted microsurgical resection of SCCM in our neurosurgical department between February 2004 and June 2020. Median age was 39 years (range 16–69 years) with an equal gender distribution. Ninety-two percent of the patients suffered from posterior column dysfunction, 38% from motor deficits, 38% had axial pain, 21% had radicular pain, 25% developed vegetative symptoms, 42% showed gait disturbance, and 21% spasticity; one patient with a SCCM localized in the cervical spine at C1 developed progressive singultus before admission. Fifty-three percent of our patients presented with an acute clinical course, and 47% showed a slowly progressive clinical course.

### Radiological findings

Initial diagnosis of SCCM was made in all patients through MRI. Mean cavernoma size was 11.3 mm (± 6.45 mm). Cavernomas were most commonly located in the cervical (*n* = 8) and the thoracic spine (*n* = 9). Brain MRI detected simultaneous supratentorial cavernomas in 2 patients. T2-signal intensity was isointense in 15%, hypointense in 31%, and hyperintense in 54%. In axial planes, position of the SCCM was ventral in one, dorsal in 10, central in 5 patients, and mostly extramedullary in one patient. In nine patients (29%), perifocal edema could be detected; three patients (13%) had a syrinx.

Complete resection of the cavernoma was confirmed in all patients via MRI before discharge or at follow-up.

### Clinical characteristics and postoperative and long-term outcome

At admission, McCormick score was 1 in five patients, 2 in eight patients, and 3 in four patients.

At discharge (mean 11 days (± 6 days)), McCormick score was 1 in 3 patients, 2 in ten patients, 3 in three patients, and 4 in one patient.

At 1-year follow-up examination, McCormick score was 1 in nine patients, 2 in five patients, and 3 in three patients. Compared to the MCS at admission, the MCS at 1-year follow-up was unchanged in 12 patients, improved in five patients, none worsened, compared to their functional status at admission. None of the patients suffered from a SCCM rebleeding event during long-term follow-up.

### IONM performance and its association with immediate and long-term outcome

Transient decrease in MEP amplitudes > 80% from baseline in all muscles of the right lower extremity were registered in one patient during cavernoma resection (case 16); the patient was neurologically unchanged at discharge and during the follow-up period of 1 year. In case 17, transient MEP loss in the muscles of the left upper extremity during cavernoma resection was associated with a new paresis of the left upper extremity at discharge which fully recovered within 3 months. In the same patient, transient 50% decrease in Tibialis-SSEP-amplitude was registered during cavernoma resection; his sensory function was unaffected.

Permanent MEP amplitude reduction > 80% from baseline was registered in three patients. In case 9, this was recorded in the left Tibialis-MEP and followed by a worsening of the paresis at discharge that still persisted at 1-year follow-up. In case 14, this was recorded in the muscles of the left lower extremity (extensor hallucis and tibialis anterior). The patient was neurologically unchanged at discharge and was improved at follow-up. In case 16, this was recorded in the muscles of the right hand but no paresis was associated postoperatively. In case 12, loss of MEP of the left deltoid muscle during cavernoma resection was not associated to a postoperative paresis.

In case 6, permanent 50% amplitude reduction of SSEP of the left leg during cavernoma resection led to a new hypesthesia of the left leg that slowly improved but was still detectable after 3 years; the patient was able to walk without external aid. Permanent 80% SSEP amplitude reduction on the left side during myelotomy led to hypesthesia (> 3 month) that after 1 year changed to dysesthesia in form of tingling sensation (case 16). In case 17, permanent median nerve-SSEP loss during cavernoma resection was associated with permanent worsening of hypesthesia (> 1 year) of the left upper extremity.

### Diagnostic test performance

In order to calculate the “predictive value” of IONM, we had to identify which IONM changes were critical and to which time point postoperatively they should be compared to (at discharge, 3-month follow-up, 1-year follow-up). Since the permanent MEP changes in our cohort were mostly an > 80% amplitude decrease or complete loss, we compared the association of permanent loss of MEP (“all or nothing” criterion) and permanent 80% decrease in amplitude with long-term neurological deficits at 1-year follow-up (we documented neurological recovery up to 1 year postoperatively). We found that the “all or nothing” criterion was not able to identify a permanent deficit in one patient while it reduced the number of false positive patients (*n* = 3 for the 80% amplitude reduction criterion, *n* = 1 for the “all or nothing” criterion).

Since the permanent decrease of 80% in amplitude in at least one muscle was sufficiently sensitive to detect all permanent deficits after long-term follow-up of 1 year, we used this criterion for diagnostic test analysis (Table 2) and surrogacy analysis (Table 3).

**Table 2:** IONM diagnostic test performance

IONM	Discharge		3-month follow-up		1-year follow-up	
	MEP	SSEP	MEP	SSEP	MEP	SSEP
TP (*n*)	1	3	1	2	1	2
TN (*n*)	4	14	8	13	8	14
FP (*n*)	3	0	3	0	3	1
FN (*n*)	4	1	0	2	0	1
Sensitivity (%)	0.25	0.75	1	0.6	1	0.67
Specificity (%)	0.5	1	0.73	1	0.73	0.93
PPV (%)	0.2	1	0.25	1	0.25	0.67
NPV (%)	0.57	0.93	1	0.87	1	0.93
Likelihood ratio	0.47	23.3	2.59	14.5	2.59	9.57

*IONM* intraoperative neurophysiological monitoring, *MEP* motor-evoked potential, *SSEP* somatosensory evoked potential, *TP* true positive, *TN* true negative, *FP* false positive, *FN* false negative, *PPV* positive predictive value, *NPV* negative predictive value

**Table 3:** Surrogacy analysis for MEP and SSEP

	MEP	SSEP
Reversible risk	0.17	0.25
Irreversible risk	0.3	0.63
Relative risk CI, 95	0.56 (0.23–1.37)	0.4 (0.18–0.89)

*CI* confidence interval, *MEP* motor-evoked potential, *SSEP* somatosensory evoked potential

Permanent SSEP changes ranged from 50% amplitude reduction to complete loss of SSEP. We tested three different warning criteria for SSEP monitoring and found the 50% amplitude reduction to be more sensitive than the other warning criteria while having the same specificity.

To determine the likelihood of new postoperative deficits, we compared their occurrence in relation to SSEP and MEP changes with the applied warning criteria asking the following question: If at the end of surgery the amplitude of MEP or SSEP are permanently reduced below 80% or 50%, respectively, what is the likelihood of a new postoperative deficit at discharge, 3-month, or 1-year follow-up compared to the likelihood of a new postoperative deficit if the amplitude remains above the predefined cut-off value? When critical MEP changes occurred, the likelihood ratio of a motor deficit 3 months and 1 year after surgery was 2.59; when critical SSEP changes were registered, the likelihood ratio of sensory deficit was 14.5 and 9.57, respectively.

### Association of reversibility of IONM deterioration to neurological outcome

Following corrective actions as described above in response to MEP and/or SSEP deterioration during resection, amplitude reductions were reversible in two respectively one case.

For the evaluation of MEP and SSEP deterioration as surrogate parameters, we analyzed the association of reversible and irreversible changes to neurological status at 1-year follow-up (Table 3). A reversible IONM change was associated with a reduced risk of a permanent neurological deterioration at 1-year follow-up compared to an irreversible IONM change (for MEP and SSEP 17 vs 30% and 25 vs 62.5%, respectively). The relative risk for a neurological deficit after 1-year follow-up following a reversible significant MEP or SSEP deterioration compared to an irreversible IONM deterioration was 0.56 (0.23–1.37) and 0.4 (0.18–0.89), respectively (Table 3).

## Discussion

Even though recently published studies [2, 7, 28, 31] suggest low surgical risk for resection of SCCM, the variability between single-center studies is high: Deutsch et al. (2010) reported in 5 out of 5 patients new dorsal column dysfunction after median myelotomy and cavernoma resection and worsening of McCormick score by one point in 4 out of 5 patients immediately after surgery [6]. Park et al. (2009) report no improvement of sensory symptoms after surgery in their cohort of 14 patients. After an average of 55-month follow-up examination, three out of 14 patients showed aggravation of motor dysfunction and in two cases worsening of dorsal column dysfunction was reported [29]. Badhiwala’s meta-analysis reported transient postoperative complications in 27.2% of patients; at follow-up, neurological function was improved in 51.5%, unchanged in 37.8%, and worsened in 10.7%. Interestingly, no differences were seen for superficial cavernomas compared to deep-seated lesions. Significant predictor of outcome was surgery within three months from onset of symptoms [3].

Our findings are in line with these literature data: at discharge in three patients (18%), functional deterioration with worsening of MCS of one point was registered. After 1 year, no patient had worse neurological function compared to their neurological status at admission. Interestingly, patients functionally improved beyond 3 months postoperatively (Table 4). On the other hand, two patients developed new pain syndromes at long-term follow-up in the form of tingling or burning sensation, which had not occurred 3 months after surgery. Both patients had complained about hypesthesia postoperatively, with improvement by the time of follow-up at 1 year. In this particular patient, these dysesthesias were not regressed even at 5-year follow-up. The persistence of pain syndromes in long-time follow-up has been previously described and may be associated to hemosiderin around the dorsal horn of spinal laminae initiating pain pathways [13].

**Table 4:** McCormick score at admission, discharge, and follow-up

	McCormick score			
	1 (*n*)	2 (*n*)	3 (*n*)	4 (*n*)
Admission	5	8	4	0
Discharge	3	10	3	1
3-month FU	6	8	3	0
1-year FU	9	5	3	0

*FU* follow-up, *n* number of patients

The inherent risk for neurological deterioration in intramedullary SCCM surgery demands for the utilization of IONM modalities. Previous studies have already analyzed the diagnostic value of IONM for surgery of different intramedullary spinal cord tumor entities (IMSCT) [5, 10, 11, 15]. These studies far mostly comprise data about infiltrating lesions which do not show a clear tumor-spinal cord interface/dissection plane, like astrocytoma or ependymomas, and in order to achieve maximum safe resection, IONM could be crucial. Due to their different biology, the resection of vascular lesions, like SCCM or hemangioblastomas, have to be considered separately from resection of infiltrating intramedullary tumor lesions and a simple transfer of data on the significance of IONM for IMSCT resection towards SCCM resection is not judicious. Compared to infiltrating lesions, where complete resection is sometimes not possible, SCCM often show a clear dissection plane. Moreover, resection of SCCM should aim for complete removal whenever possible, as incomplete removal does not appear to eliminate the risk of rebleeding. As such, the decision for termination of SCCM surgery due to IONM changes anticipating permanent neurological deficit needs a solid ground demanding critical and separate (from other IMSCT entity) analysis. Although several studies have now reported the clinical outcome of surgically treated patients with SCCM [1, 3, 28, 38], we here report about one of the rare corresponding case series describing microsurgical SCCM resection with stringent utilization of multimodal IONM including both SSEP and MEP—as generally recommended in IMSCT surgeries [35, 39, 41]. Only the study of Li et al. (2019) is also reporting about patients who underwent surgery for SCCM with MEP monitoring being available in 52 cases and SSEP monitoring in 32 cases [21]. However, the authors of this study analyze the ability of critical MEP and SSEP changes in those patients to predict postoperative functional changes using only a compound functional score (MCS), but without precise description of postoperative neurological deficits in detail. Though the authors are following another definition of “true positive changes,” that in our series demands that the neurological change matches the modality of IONM (for example, MEP changes and postoperative paresis).

With regard to IONM warning criteria, the 50% amplitude reduction and/or an increase in SSEP latency ≥ 2 ms are well-established glial/ependymal IMSCT surgery [33, 36], while the definition of warning criteria for MEP in the absence of D-wave monitoring is more difficult and, e.g., complete loss of MEP, have been suggested in glial/ependymal IMSCT surgery [17, 23]. However, for deformity surgery as well as for spinal cord vascular lesions, like hemangioblastomas, case series have also suggested a MEP warning criterion of an 80% amplitude reduction as optimal threshold for impending neurological deficits [17, 18, 21, 23, 36]. Since these warning criteria have never been tested for SCCM surgery, we looked at diagnostic test performance using different warning criteria (Tables 5 and 6) and found that the 50% amplitude reduction of SSEP and 80% amplitude reduction of MEP were the most accurate. An all or nothing criterion has been proposed for MEP if the D-wave is monitorable, but in our cases, surveying without D-wave monitoring would have reduced the sensitivity of MEP [24].

**Table 5:** Association of MEP deterioration with clinical outcome using two different criteria for the definition of a positive IONM-event

		Neurological deficit		
		+	−	
80% amplitude reduction	+	1	3	Sens = 1 Spec = 0.73
	−	0	8	
“all or nothing”	+	0	1	Sens = 0 Spec = 0.91
	−	1	10	

*Sens* sensitivity, *Spec* specificity

**Table 6:** Association of SSEP-deterioration with clinical outcome using three different criteria for the definition of a positive IONM-event

		Neurological deficit		
		+	−	
50% amplitude reduction	+	2	1	Sens = 0.67 Spec = 0.93
	−	1	14	
80% amplitude reduction	+	1	1	Sens = 0.33 Spec = 0.93
	−	2	14	
“all or nothing”	+	1	1	Sens = 0.33 Spec = 0.93
	−	2	14	

*Sens* sensitivity, *Spec* specificity

Due to the high number of false-positive MEP deteriorations (*n* = 3), the association of permanent MEP deterioration and postoperative paresis was not significant (*p* = 0.333 at 1-year follow-up). However, transient MEP deterioration was never followed by permanent paresis at 3-month and 1-year follow-up in our series, confirming previous reports [16]. While all patients were able to walk independently (one patient with braces) at 1-year follow-up, we could show in our case series that even irreversible deterioration of MEP amplitude, and not only MEP amplitude loss, might also be followed with long-term partial motor dysfunction exceeding the information provided in previous IMSCT studies.

On the other hand, critical MEP amplitude reduction which is always followed by corrective maneuvers is named a false-positive event if the patient does not show a neurological worsening, but might as well have been a true positive if no corrective maneuvers would have been engaged. This is why the surgeon intraoperatively has to consider the plausibility of IONM changes.

Intraoperative IONM changes can also be classified as “expected” if due to location or trajectory to access the cavernoma a corresponding IONM change during surgery occurs and is therefore plausible. The “unexpected” IOM changes are a challenge as those point more likely towards technical or general causes. In case of implausibility, the termination of surgery is not mandatory. A distinction can also be made for the dynamics of IONM changes. It has been an observation during IMSCT surgery that a sudden loss of MEP/SSEP rather indicates a vascular problem when there is no obvious disruption of fiber tracts, while a more stepwise reduction of MEP/SSEP amplitude points towards either traction or technical reasons. In our case collection, we were not able to demonstrate these observations. The only sudden loss of MEP occurred in patient No. 12 who presented with hemiparesis preoperatively. The cavernoma was centrally located in C3/4. During tumor resection, when sudden loss of MEP was registered, all corrective maneuvers as described in the “Methods” section were applied, since too much traction or even a vascular reason were possible. The MEP did not recover, but the surgical team considered a vascular reason unlikely completed resection of the cavernoma. At discharge, the patient showed no worsening of paresis and regained complete motor function within 1 year (supplemental Table1).

SSEP deterioration during surgery was significantly associated with postoperative sensory impairment at discharge (*p* = 0.0049) and 3-month follow-up (*p* = 0.0123). With patients recovering from a transient neurological deficit (*n* = 2), the association at 1-year follow-up was not significant (*p* = 0.0564).

A practical limitation of SSEP monitoring during surgery is the duration needed for averaging (minimum of 30 s, depending on the signal-to-noise-ratio) and therefore information of impending injury is brought to the surgeon with a delay. This in consequence can delay also corrective actions. Furthermore, SSEP assess highly specific the conductivity of the dorsal columns, but do not monitor the spinothalamic tract. As such, progressive dysesthesia was documented in two patients of our cohort, even though epicritic sensibility and motor function stayed intact. Since dysesthesia was progressive over several months, it may have been due to gliosis affecting the spinothalamic tract. We would like to emphasize that SSEP monitoring is very important, because paresthesias in particular are described by patients as very unpleasant and impairing. Not infrequently, this leads to incapacity to return to work.

D-wave monitoring improves monitoring of the corticospinal tract during spinal cord surgery and has proven to be the best surrogate for long-term outcome [24, 37]. In our retrospective analysis of SCCM surgery, D-wave monitoring was employed since 2016 in a total of 6 cases, while 2 were not monitorable. Since only 4 cases in our study had a reliable D-wave monitoring, we did not perform a separate analysis of D-wave monitoring during resection of SCCM, but only report that in all 4 cases a stable D-wave was registered and associated to no long-term motor deficit (supplemental Table 1).

Rather than predict a neurological deficit, IONM should be aimed to warn the surgeon of an impending neurological deficit. Diagnostic tests omit to evaluate reversibility of IONM changes which may be achieved by immediate initiation of corrective surgical maneuvers in a standardized fashion. However, surrogacy analysis takes reversible IONM changes into account and evaluates their effect on clinical outcome [9]. Supplemental Table 1 shows that most of IONM changes occurred during tumor resection and were “expected” and therefore accessible to corrective actions. The relative risk reports if IONM recovery correlates with better neurological outcome compared to irreversible IONM deterioration. The RR for both SSEP and MEP in our study indicates a better neurological outcome if IONM changes were reversible, even though it is not possible to discriminate which maneuver of the corrective actions in particular can offer the highest probability of reversibility of IONM changes in a retrospective analysis. Moreover, due to low number of reversible IONM deteriorations, only a descriptive analysis is feasible and successful corrective actions without IONM recovery are not registered here. Therefore, further prospective studies are warranted to elucidate this issue.

Nevertheless, our data show that IONM is feasible to give fast feedback on potential harmful cord manipulation, e.g., traction or compression, in SCCM resection. The relationship between reversible IONM changes and a more favorable prognosis with regard to neurological outcome alludes to the importance of IONM—and the evaluation of this relationship in SCCM surgery is a major importance of our study compared to other SCCM case series like the study of Li et al. (2019) that only calculate sensitivity and specificity values with regard to permanent IONM changes and postoperative status changes [21]. With drawing attention to the concept of potentially reversible IONM changes, step-by-step actions can be initiated immediately in the event of MEP/SSEP changes to allow restoration of IONM parameters assuming this will reduce the risk of avoidable neurological deficits. At this stage of research, we would like to recommend the following stepwise pragmatic approach for immediate intraoperative corrective actions according to Sala et al. (2007) which in our opinion increases the chance for SSEP or MEP recovery [34].

Based on our data, termination of resection might be considered if corrective maneuvers are not followed by recovery of MEP and further MEP deterioration occurs during careful resumption of resection outbalancing gains and risks of a subtotal resection versus a potentially severe and permanent postoperative deficit.

Limitations of this study are the small number of patients as well as the retrospective character of the study. On the other hand, SCCM is a rare disease and this is the first detailed description of IONM during SCCM resection with detailed neurological long-term follow-up examinations.

## Conclusions

In this study, we can confirm microsurgical resection with multimodal IONM to be a safe method to treat SCCM and eliminate the risk of rebleeding. Even though studies have looked at the role of IONM for surgery of IMSCT of different pathologies, the role of IONM during resection of SSCM has not been analyzed so far. A decrease of 80% of MEP amplitude or 50% of SSEP amplitude, respectively, which persisted until the end of surgery, performed as good warning criteria in our study. Reversibility of MEP and SSEP changes showed an association with better neurological outcome compared to irreversible IONM deterioration. Further studies, especially prospective studies are necessary to analyze the association of corrective maneuvers and electrophysiological as well as neurological recovery.

## Supplementary Information

Below is the link to the electronic supplementary material.

Supplementary file1 (XLSX 15 KB)

## Abbreviations

MEP: Motor-evoked potential
SSEP: Somatosensory-evoked potential
IONM: Intraoperative neurophysiological monitoring
SCCM: Spinal cord cavernous malformation
IMSCT: Intramedullary spinal cord tumor
RR: Relative risk
